# Dysregulated meta-organismal metabolism of aromatic amino acids in alcohol-associated liver disease

**DOI:** 10.1097/HC9.0000000000000284

**Published:** 2023-10-12

**Authors:** Marko Mrdjen, Emily Huang, Vai Pathak, Annette Bellar, Nicole Welch, Jaividhya Dasarathy, David Streem, Craig J. McClain, Mack Mitchell, Svetlana Radaeva, Bruce Barton, Gyongyi Szabo, Srinivasan Dasarathy, Zeneng Wang, Stanley L. Hazen, J. Mark Brown, Laura E. Nagy

**Affiliations:** 1 Department of Inflammation and Immunity, Cleveland Clinic, Cleveland, Ohio, USA; 2 Department of Cardiovascular and Metabolic Sciences, Cleveland Clinic, Cleveland, Ohio, USA; 3 Department of Cancer Biology, Cleveland Clinic, Cleveland, Ohio, USA; 4 Department of Gastroenterology and Hepatology, Cleveland Clinic, Cleveland, Ohio, USA; 5 Department of Family Medicine, Metro Health Medical Center, Cleveland, Ohio, USA; 6 Department of Psychiatry and Psychology, Cleveland Clinic Lutheran Hospital, Cleveland, Ohio, USA; 7 Department of Medicine, University of Louisville, Louisville, Kentucky, USA; 8 Internal Medicine, University of Texas Southwestern Medical Center, Dallas, Texas, USA; 9 National Institute on Alcohol Abuse and Alcoholism, Bethesda, Maryland, USA; 10 Department of Population and Quantitative Health Sciences, University of Massachusetts Medical School, Worcester, Massachusetts, USA; 11 Department of Medicine, Beth Israel Deaconess Medical Center, Harvard Medical School, Boston, Massachusetts, USA; 12 Department of Molecular Medicine, Case Western Reserve University, Cleveland, Ohio, USA; 13 Center for Microbiome and Human Health, Lerner Research Institute, Cleveland Clinic, Cleveland, Ohio, USA

## Abstract

**Background:**

Chronic alcohol consumption impairs gut barrier function and perturbs the gut microbiome. Although shifts in bacterial communities in patients with alcohol-associated liver disease (ALD) have been characterized, less is known about the interactions between host metabolism and circulating microbe-derived metabolites during the progression of ALD.

**Methods:**

A large panel of gut microbiome-derived metabolites of aromatic amino acids was quantified by stable isotope dilution liquid chromatography with online tandem mass spectrometry in plasma from healthy controls (n = 29), heavy drinkers (n = 10), patients with moderate (n = 16) or severe alcohol-associated hepatitis (n = 40), and alcohol-associated cirrhosis (n = 10).

**Results:**

The tryptophan metabolites, serotonin and indole-3-propionic acid, and tyrosine metabolites, p-cresol sulfate, and p-cresol glucuronide, were decreased in patients with ALD. Patients with severe alcohol-associated hepatitis and alcohol-associated cirrhosis had the largest decrease in concentrations of tryptophan and tyrosine-derived metabolites compared to healthy control. Western blot analysis and interrogation of bulk RNA sequencing data from patients with various liver pathologies revealed perturbations in hepatic expression of phase II metabolism enzymes involved in sulfonation and glucuronidation in patients with severe forms of ALD.

**Conclusions:**

We identified several metabolites decreased in ALD and disruptions of hepatic phase II metabolism. These results indicate that patients with more advanced stages of ALD, including severe alcohol-associated hepatitis and alcohol-associated cirrhosis, had complex perturbations in metabolite concentrations that likely reflect both changes in the composition of the gut microbiome community and the ability of the host to enzymatically modify the gut-derived metabolites.

## Introduction

Alcohol-associated liver disease (ALD) is one of the most prevalent liver diseases in the world and represents a global health burden.^1^ ALD encompasses several pathologies that range from mild steatosis to more harmful diagnoses, such as fibrosis, cirrhosis, and alcohol-associated hepatitis (AH), with some patients developing HCC.^2^ Although chronic alcohol consumption is the main factor behind the progression of ALD, other contributors such as sex, ethnicity, obesity, and genetic factors can also influence the course of the disease.^3^ Patients can progress to more severe disease, when end-stage interventions, such as liver transplants, are required. Furthermore, as there is a lack of effective therapeutics, mortality remains high in patients with severe forms of ALD.^4^ Chronic, heavy consumption of alcohol disrupts gut barrier function and alters the gut microbiome community.^5^ The prevailing theory is that patients with ALD are more susceptible to increased liver inflammation, at least in part due to the increased translocation of bacterial byproducts from the intestines to the liver through the portal vein.

The gut microbiome plays key roles in metabolism, contributing to the maintenance of gut barrier integrity, and modulating intestinal immune responses.^6^ Moreover, since many microbial metabolites enter the circulation, the gut microbiome can also directly impact systemic and organ immune responses.^7,8^ The changes in gut microbial composition during the progression of ALD are just beginning to be understood; in particular, more work is needed to identify how gut microbial metabolites are altered in ALD.^9^ Prior research has demonstrated that key metabolites that regulate host physiology, such as short-chain fatty acids, indole derivatives, and choline-derived metabolites, are impacted by ethanol administration.^10,11^ The most well-studied metabolites are tryptophan-derived indole derivatives, which are decreased in patients with ALD.^10,12^ Exogenous provision of these metabolites is effective in reducing liver injury in murine models of ALD.^9,10,12^ Indole metabolites that activate the aryl hydrocarbon receptor (AHR) are capable of enhancing the gut barrier and modulating the intestinal immune response to prevent the leakage of bacterial endotoxin to the liver. Alternatively, inhibition of bacterial production of choline-associated gut microbial metabolites, such as trimethylamine (TMA), also effectively blunts ethanol-induced liver damage in mice.^11^ Indeed, the data on choline-associated metabolites highlight the metabolism-dependent interplay between microbes and host metabolism, and its relevance in ALD. This cross talk between microbes and the host is known as meta-organismal metabolism, and these microbial metabolic circuits could provide novel treatments for ALD.^11^ Thus, further identification and validation of gut microbial metabolites perturbed in ALD are essential for developing novel therapeutics to prevent the progression of disease in patients with ALD.

Previous studies have used untargeted metabolomics approaches to investigate alterations in the stool metabolome from patients with ALD, and more recent targeted (stable isotope dilution) metabolomics approaches have used quantitative measures of multiple structurally distinct microbial and meta-organismal metabolites to identify pathways that are linked to disease risks and adverse outcomes.^12,13^ Here, we used a targeted metabolomics approach focused on gut microbe-derived metabolites from aromatic amino acids to evaluate differences in concentrations in the plasma of healthy controls (HCs), patients classified as heavy drinkers (HDs), patients with moderate (mAH) or severe alcohol-associated hepatitis (sAH), and patients with alcohol-associated cirrhosis (AC). Importantly, as many gut microbial metabolites are metabolized in the liver, we investigated perturbations in this meta-organismal interaction by assessing the expression of hepatic enzymes associated with the metabolism of specific gut microbial metabolites found to be impacted by ALD, focusing on enzymes involved in phase II metabolism and tryptophan metabolism.

## Methods

### Patient cohorts

Deidentified plasma samples from a total of 29 HCs, 10 HDs, 10 patients with AC, and 10 patients with mAH were obtained from the Northern Ohio Alcohol Center (NCT03224949). In addition, samples from 40 patients with sAH were obtained from the Defeat Alcoholic Steatohepatitis trial along with an additional 6 patients with mAH (NCT018909132). In this randomized multicenter trial, patients with AH were recruited from the Cleveland Clinic, the University of Massachusetts Medical School, the University of Texas Southwestern Medical Center, and the University of Louisville School of Medicine. Patients were stratified based on disease severity according to the MELD score. Patients with sAH were defined as having MELD ≥20, while mAH was defined as patients with MELD <20. A detailed description of the Defeat Alcoholic Steatohepatitis clinical trial and outcomes has been described.^14,15^ In this trial, patients were tracked over a period of 180 days following treatment; however, in the current analysis, only samples from the time of admission, ie, before treatment, were analyzed. Information pertaining to diet or antibiotic use was not collected from any of the subjects included in the study. All research was conducted in accordance with both the Declarations of Helsinki and Istanbul. Study approval was obtained from the Institutional Review Boards of all 4 participating institutions, and informed consent was obtained from all participants prior to the collection of plasma samples.

### Liquid chromatography–mass spectrometry quantification of aromatic amino acid metabolites

The aromatic amino acid metabolites were quantified by stable isotope dilution liquid chromatography–mass spectrometry analysis using methods recently described in detail.^13^ The TMA-related metabolites were quantified by stable isotope dilution liquid chromatography–mass spectrometry analysis as reported^16^

### RNA sequencing analysis from patients

RNA sequencing (RNA-seq) data from patients with different etiologies of liver disease were obtained from Argemi et al^17^ (dbGAP phs001807.v1.p1). Fastq files were aligned to the human genome (GRCh38, release 96) with 100 bootstraps calculated. Differential gene expression was calculated with a false discovery rate cutoff of *q* < 0.05. For the boxplots and heatmaps in this study, bootstraps were converted to transcripts per million. For the meta-analysis, additional RNA-seq data were obtained from Massey et al^18^ (GSE142530) and Hyun et al^19^ (GSE143318), and combined with the Argemi and colleagues, data set. Here, we applied a random effects’ model on individual fold changes, while a Bonferroni adjustment was used to obtain adjusted *p*-values.

### Statistical analysis

All values depicted are represented as means ± SEM. Significant differences between values were tested by ANOVA using the general linear model with a Tukey *post hoc* test based on results obtained from the Kolmogorov-Smirnov normality test. *p*-Values < 0.05 were used to determine significant differences between groups. In the boxplots, figures with different alphabetical subscripts are significantly different, while shared subscripts indicate no significance. For the initial heatmap, log fold change was calculated between the average concentrations of each ALD group to that of HCs for each metabolite. Heatmaps for RNA-seq data were generated by log-transformed transcripts per million with no row and sample clustering. Differential gene expression was tested with boxplots comparing the transcripts per million of each group along with *q* values indicated significance based on the false discovery rate. Spearman correlation coefficient was used to analyze the association of microbe-derived metabolites and clinical features of interest.

### Human samples for immunoblotting

Explant tissue from patients with sAH and HCs was obtained from the Clinical Resource for Alcohol-associated Hepatitis Investigations conducted at Johns Hopkins University (R24 AA0025107, Z. Sun PI). Informed consent was obtained from each patient prior to collection, and study approval was obtained from the Institutional Review Board at Johns Hopkins University. Whole tissue lysates were made in a modified RIPA buffer before proteins were separated on a 4%–12% SDS-PAGE gel before being transferred to polyvinylidene difluoride membranes.^11^ Proteins for sulfotransferase (SULT) family 1A member 1 (10911-2-AP; Thermo Fisher, Waltham, MA) and UDP glucuronosyltransferase (UGT) family 1 member A6 (PA5-22319; Thermo Fisher, Waltham, MA) were detected after incubation with specific antibodies.

## Results

### Patient characteristics

Patient demographics and clinical data are summarized in Table 1. For this study, both males and females were included; however; there was a difference in the male/female ratios between the HC and sAH groups. The study population was predominantly white with the exception of patients in the HD group, who were 50% African American. As expected, the MELD score, aspartate aminotransferase, bilirubin, international normalized ratio, and the Child-Pugh score were all highest in patients with sAH. Patients diagnosed with AC had the highest concentrations of albumin and creatinine compared to the other groups.

**TABLE 1:** Demographic and clinical characteristics of study participants

	HC (N = 29)	HD (N = 10)	mAH (N = 16)	sAH (N = 40)	AC (N = 10)
Age					
Mean	46.28	44.50	48.06	44.88	49.30
Range	23–66	29–63	26–60	25 – 65	35–66
Sex, n (%)					
Female	18 (62.1)	5 (50.0)	8 (50.0)	17 (42.5)	5 (50.0)
Male	11 (37.9)	5 (50.0)	8 (50.0)	23 (57.5)	5 (50.0)
Race, n (%)					
Black	5 (16.7)	5 (50.0)	1 (16.2)	2 (5.0)	1 (10.0)
Asian	2 (6.7)	0 (0.0)	0 (0.0)	0 (0.0)	0 (0.0)
Hispanic	1 (3.3)	0 (0.0)	0 (0.0)	0 (0.0)	0 (0.0)
Unknown	4 (13.3)	1 (10.0)	0 (0.0)	1 (2.5)	0 (0.0)
White	18 (60.0)	4 (40.0)	15 (93.8)	37 (92.5.0)	9 (90.0)
MELD					
Mean (SE)	NA	NA	14.07 (1.36)	26.08 (1.60)	14.00 (2.79)
AST					
Mean (SE)	20.79 (1.63)	25.90 (2.04)	103.31 (18.95)	136.98 (11.35)	46.30 (6.82)
ALT					
Mean (SE)	20.95 (2.01)	24.00 (4.28)	57.75 (13.23)	44.25 (3.76)	26.40 (4.04)
Albumin					
Mean (SE)	4.34 (0.04)	3.97 (0.11)	2.89 (0.21)	2.53 (0.07)	3.34 (0.32)
Creatinine					
Mean (SE)	0.88 (0.05)	0.95 (0.11)	0.72 (0.06)	0.93 (0.09)	1.09 (0.23)
Bilirubin					
Mean (SE)	0.59 (0.07)	0.72 (0.09)	5.12 (1.12)	21.42 (1.17)	5.03 (2.79)
INR					
Mean (SE)	1.03 (0.02)	1.01 (0.03)	1.25 (0.06)	1.96 (0.06)	1.38 (0.10)
Child-Pugh Score					
Mean (SE)	NA	NA	8.57 (0.43)	10.75 (0.21)	8.00 (0.65)

Abbreviations: AC, alcohol-associated cirrhosis; HC, healthy control; HD, heavy drinker; mAH, moderate alcohol-associated hepatitis; sAH, severe alcohol-associated hepatitis.

### Gut-derived microbial metabolites were dysregulated in the plasma of patients at different stages of ALD

Chronic alcohol consumption is associated with changes in both the gut microbiome and hepatic metabolism, both of which are associated with disease progression.^5,20^ Therefore, we quantified changes in the concentrations of gut microbe-derived metabolites at different stages of ALD using a gut microbial metabolite panel representing physiologically relevant metabolites related to the metabolism of tryptophan, phenylalanine, TMA, and tyrosine. This panel not only contains metabolites produced directly by the gut microbiome but also several dietary precursors for microbial production of metabolites including choline, betaine, carnitine (precursors for TMA), and tryptophan (precursor for indoles). The metabolites within this panel are implicated in the pathogenesis of multiple chronic diseases, including inflammatory bowel disease, cardiovascular disease, and chronic kidney disease.^13,21–24^ This approach allowed us to directly measure and compare concentrations of selected metabolites between groups to investigate how key metabolites that regulate host physiology are altered with ALD.

**FIGURE 1:**
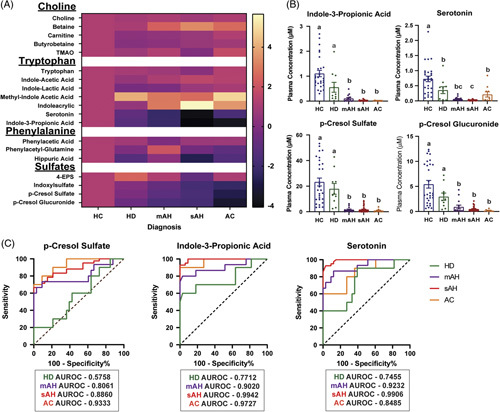
Gut-derived microbial metabolites are dysregulated in the plasma of patients in various stages of the alcohol-associated liver disease (ALD) spectrum. (A) Heatmap of metabolite concentrations in patients with ALD compared to healthy controls. Log fold change was measured for HD (n = 10), patients with mAH (n = 16), sAH (n = 40), and AC (n = 10) and compared to HC (n = 29). (B) Concentrations of indole-3-propionic acid, serotonin, p-cresol sulfate, and p-cresol glucuronide were measured in the plasma of patients with ALD using liquid chromatography with tandem mass spectrometry. (C) ROC curves for distinguishing patients with ALD from healthy controls. AUROC for each group is illustrated. Values represent means ± SEM. Statistical analysis was conducted in SAS; values with different superscripts are significantly different (*p* < 0.05). Abbreviations: 4-EPS, 4-ethylphenyl sulfate; AC, alcohol-associated cirrhosis; ALD, alcohol-associated liver disease; HC, healthy controls; HD, heavy drinkers, mAH, moderate alcohol-associated hepatitis; sAH, severe alcohol-associated hepatitis; TMAO, trimethylamine N-oxide.

Decreased concentrations of specific gut microbial metabolites analyzed were most prevalent in patients with mAH, sAH, and AC, but not HD, compared to HC; the most heavily affected classes of metabolites were tryptophan and sulfate-conjugated microbial metabolites (Figure 1A, B). While most microbial metabolites were decreased in patients with ALD, some of the less abundant tryptophan metabolites, including methyl-indole acetic acid and indoleacrylic acid, and betaine, a dietary TMA precursor, were increased in patients with ALD (Table 2, Supplemental Figure S1, http://links.lww.com/HC9/A560).

**TABLE 2:** Median metabolite concentrations and ranges

	HC	HD	mAH	sAH	AC
Metabolite	Median (IQR) µM	Median (IQR) µM	Median (IQR) µM	Median (IQR) µM	Median (IQR) µM
Choline	10.50 (7.134; 24.05)	30.97 (22.10; 35.26)	26.60 (20.68; 37.70)	10.59 (8.10; 26.61)	33.47 (22.47; 36.37)
Betaine	40.46 (30.59; 97.73)	132.6 (73.77; 227.1)	236.7 (122.7; 443.5)	452.6 (202.1; 740.2)	327.5 (144.2; 730.1)
Carnitine	26.26 (19.38; 29.66)	28.39 (24.59; 32.66)	29.29 (24.59; 37.72)	33.49 (24.44; 48.82)	43.68 (33.36; 87.34)
Butyrobetaine	0.74 (0.63; 0.93)	0.72 (0.49; 0.93)	0.72 (0.61; 1.22)	0.99 (0.77; 1.51)	0.95 (0.74; 1.94)
Trimethylamine N-oxide	3.35 (1.78; 10.04)	9.62 (5.52; 17.30)	5.99 (0.72; 8.42)	0.56 (0.21; 2.89)	0.59 (0.26; 4.66)
Tryptophan	119.4 (101.1; 304.2)	368.0 (290.0; 513.4)	257.1 (128.8; 364.7)	71.14 (34.21; 132.3)	484.4 (262.9; 650.0)
Indole acetic acid	1.59 (1.28; 2.86)	2.65 (1.75; 3.42)	1.80 (1.23; 2.68)	2.20 (0.94; 4.94)	2.50 (1.23; 5.61)
Indole-lactic acid	0.98 (0.68; 1.19)	0.57 (0.46; 0.80)	0.35 (0.27; 0.48)	0.70 (0.39; 1.72)	0.80 (0.52; 1.28)
Methyl-indole acetic acid	0.007 (0.003; 0.012)	0.03 (0.01; 0.03)	0.04 (0.02; 0.11)	0.01 (0.004; 0.064)	0.25 (0.04; 0.74)
Indoleacrylic acid	0.08 (0.04; 0.11)	0.07 (0.04; 0.17)	0.14 (0.08; 0.22)	0.14 (0.07; 0.36)	0.19 (0.08; 0.64)
Serotonin	0.71 (0.31; 0.94)	0.36 (0.03; 0.51)	0.04 (0.02; 0.09)	0.02 (0.006; 0.03)	0.06 (0.02; 0.35)
Indole-3-propionic acid	0.86 (0.65; 1.34)	0.25 (0.11; 1.04)	0.02 (0.007; 0.17)	0.008 (0.006; 0.016)	0.004 (0.002; 0.01)
Phenylacetic acid	0.58 (0.03; 1.03)	1.19 (0.69; 1.90)	0.54 (0.31; 1.00)	0.33 (0.01; 0.87)	1.27 (0.62; 1.92)
Phenylacetyl glutamine	1.87 (0.93; 2.55)	1.68 (0.71; 2.65)	0.56 (0.21; 2.13)	0.50 (0.25; 1.16)	0.71 (0.52; 1.19)
Hippuric acid	6.30 (3.66; 14.50)	4.22 (2.00; 10.30)	1.85 (1.04; 3.85)	0.93 (0.49; 1.60)	1.71 (0.78; 9.43)
4-Ethylphenyl sulfate	0.24 (0.17; 0.52)	0.33 (0.21; 1.86)	0.07 (0.04; 0.37)	0.12 (0.07; 0.29)	0.13 (0.03; 0.41)
Indoxyl sulfate	3.96 (1.96; 5.91)	2.97 (2.25; 6.30)	0.93 (0.41; 1.96)	0.10 (0.01; 0.78)	0.41 (0.05; 1.64)
p-Cresol sulfate	16.88 (9.55; 38.80)	13.63 (7.20; 26.42)	0.53 (0.14; 2.90)	0.42 (0.12; 1.98)	0.18 (0.05; 1.50)
p-Cresol glucuronide	3.39 (1.95; 9.42)	2.33 (1.03; 4.52)	0.19 (0.08; 1.41)	0.23 (0.16; 0.55)	0.07 (0.03; 0.25)

Abbreviations: AC, alcohol-associated cirrhosis; HC, healthy control; HD, heavy drinker; mAH, moderate alcohol-associated hepatitis; sAH, severe alcohol-associated hepatitis.

To investigate the relationship between the severity of AH and metabolite concentrations, we carried out a correlation analysis between clinical features of patients with mAH and sAH with metabolite concentrations (Supplemental Figure S2, http://links.lww.com/HC9/A560). Although there were significant correlations between sulfate-conjugated microbial metabolites and tryptophan metabolites, based on Spearman coefficients, the strength of these correlations was weak. Receiver operating characteristic curves were designed to test if individual metabolites of interest could differentiate patients with ALD from HC (Figure 1C). P-cresol sulfate concentrations could distinguish patients with mAH, sAH, and AC, but not HDs, from HC. However, concentrations of tryptophan metabolites indole-3-propionic acid and serotonin were more sensitive and could distinguish all patients from HCs.

### Expression of hepatic enzymes involved in phase II sulfonation and glucuronidation pathways was perturbed in patients with sAH

The results of our initial metabolite screen showed that two p-cresol cometabolites, p-cresol sulfate and p-cresol glucuronide, were decreased in patients with ALD. In humans, p-cresol is uniquely produced by the gut microbiome from the metabolism of dietary tyrosine.^25^ Following production in the gut, p-cresol enters the liver where it directly undergoes phase II metabolism, leading to its sulfonation or glucuronidation, through sulfotransferases and glucuronosyltransferases, respectively, into its cometabolite forms. Phase II metabolism consists of conjugation reactions that produce more polar and less active metabolites, allowing for excretion into the urine or stool.^26^ These p-cresol cometabolites are positively associated with chronic kidney diseases;^27^ however, the glomerular filtration rate, an indicator of kidney function, in patients with sAH was only negatively correlated with 4-ethylphenyl sulfate concentrations and not with other sulfate-conjugated microbial metabolites (Supplemental Figure S3, http://links.lww.com/HC9/A560).

**FIGURE 2:**
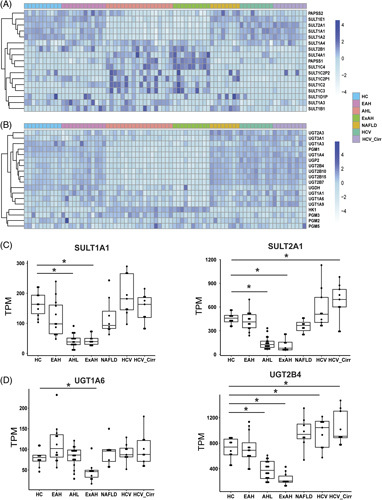
Liver expression of enzymes involved in phase II sulfonation and glucuronidation pathways are perturbed in patients with severe alcohol-associated liver disease. Heatmaps were created by log transforming TPM and grouping samples by disease categories, such as HC (n = 10), EAH (n = 12), AHL (n = 18), explant tissue from patients with sAH with liver transplant (ExAH, n = 10), NAFLD (n = 8), HCV (n = 9), and HCV_Cirr (n = 9). (A) Expression of sulfotransferases and phosphosulfate synthase enzymes that comprise the liver sulfonation pathway. (B) Expression of glucuronosyltransferases and phosphoglucomutases involved in the liver glucuronidation pathway. (C) Boxplots of SULT1A1 and SULT2A1 gene expression in TPM of RNA sequencing data from patients with various liver diseases. (D) Boxplots of UGT1A6 and UGT2B4. Error bars indicate SD, and (*) indicates *q* < 0.05. Abbreviations: AH, alcohol-associated hepatitis; AHL, AH with liver failure; EAH, early alcohol-associated hepatitis; ExAH, explant tissue from patients with severe AH after liver transplant; HC, healthy control; HCV_Cirr, HCV with cirrhosis; sAH, severe alcohol-associated hepatitis; SULT, sulfotransferase; TPM, transcripts per million; UGT, UDP glucuronosyltransferase.

We hypothesized that 1 explanation for decreased concentrations of the p-cresol cometabolites is dysregulation of critical phase II metabolism pathways in the liver. Bulk RNA-seq data of patients with liver pathologies of different etiologies revealed that both sulfonation and glucuronidation pathways were perturbed in patients with sAH (Figure 2A, B). Patients with AH with liver failure and explant tissue from patients with sAH undergoing liver transplant sAH showed unique patterns of expression of enzymes involved with phase II metabolism. Expression of 4 sulfotransferases, which transfer the sulfonate group from a donor molecule to a metabolite, was decreased in sAH groups (Figure 2A), and 3 of these sulfonation enzymes, SULT1E1, SULT2A1, and SULT1A1, belong to a class of steroid sulfotransferases. In contrast, other sulfotransferases, notably those with lower overall hepatic expression, were upregulated in sAH (Figure 2A). Hepatic expression of enzymes involved in glucuronidation was also perturbed in patients with sAH; however, more glucuronosyltransferases were decreased compared to the sulfotransferases (Figure 2B). Importantly, expression of SULT1A1, the sulfotransferase directly involved in sulfonation of p-cresol to p-cresol sulfate and indole to indoxyl sulfate, and SULT2A1, a sulfotransferase involved in estrogen and bile acid metabolism, was decreased in patients with sAH (Figure 2C). Similarly, UGT1A6, the enzyme involved in p-cresol glucuronidation, was decreased in explant tissue from patients with alcohol-associated hepatitis, while bile acid glucuronosyltransferase UDP glucuronosyltransferase family 2 member B4 UGT2B4 was decreased in the sAH groups but increased with NAFLD and HCV with cirrhosis (Figure 2D).

**FIGURE 3:**
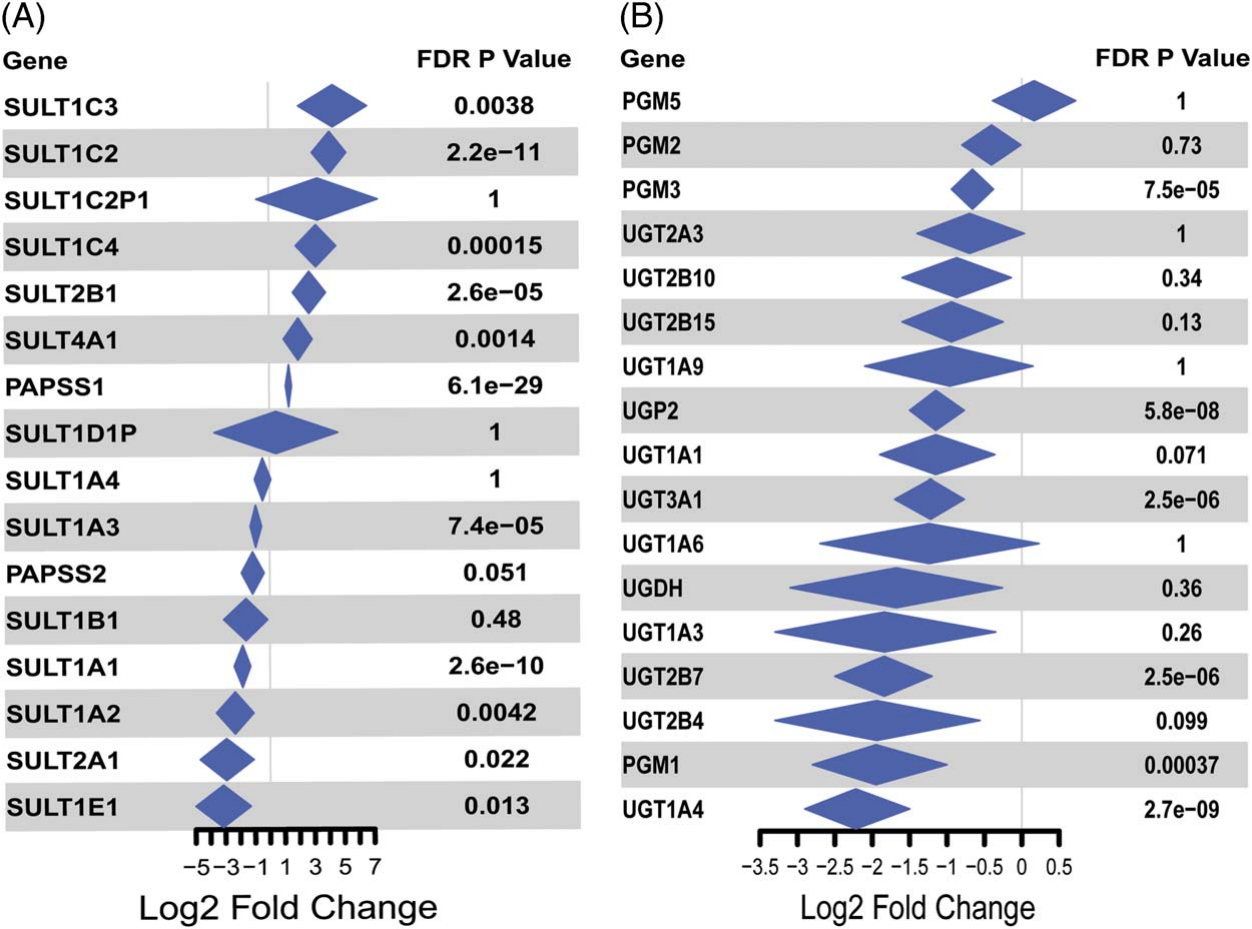
Meta-analysis reveals perturbations of phase II metabolism enzymes in 3 separate bulk RNA sequencing data sets. Violin plots were created by applying a random effects’ model on each of the individual fold changes to create the summarized diamonds, followed by a Bonferroni adjustment for *p*-values. (A) Log2 fold change, between healthy controls and sAH explant tissue, of sulfotransferases involved in the liver sulfonation pathway. (B) Log2 fold change of phosphoglucomutases involved in the liver glucuronidation pathway. Abbreviations: AH, alcohol-associated hepatitis; AHL, AH with liver failure; EAH, early alcohol-associated hepatitis; HC, healthy control; HCV_Cirr, HCV with cirrhosis; sAH, severe alcohol-associated hepatitis; SULT, sulfotransferase TPM, transcripts per million; UGT, UDP glucuronosyltransferase.

To further validate the perturbations of phase II enzymes in the liver of patients with AH, a meta-analysis was conducted using a combination of the original Argemi and colleagues RNA-seq data set with 2 additional data sets comparing HC to liver explants from patients with sAH. This analysis confirmed that steroid sulfotransferases SULT1E1, SULT1A1, SULT1A1, and SULT2A1 were decreased in sAH explant tissue, while some less highly expressed hepatic sulfotransferases were increased in these patients compared to HC (Figure 3A). These data also validated our findings of impaired phase II glucuronidation as most of the enzymes within this pathway had decreased expression in sAH (Figure 3B).

### Hepatic gene expression of enzymes involved in tryptophan metabolism was perturbed in patients with sAH

Tryptophan undergoes metabolism through 3 main pathways: the kynurenine pathway, the serotonin pathway, and the indole pathway.^28^ In humans, metabolism by means of the indole pathway mainly occurs through the gut microbiome, while serotonin and kynurenine can be metabolized by the host.^28^ Although our targeted metabolomics panel includes metabolites from the indole pathway, we hypothesized that, similar to phase II metabolism, patients with severe forms of AH have perturbations in the pathways of serotonin and kynurenine metabolism.

**FIGURE 4:**
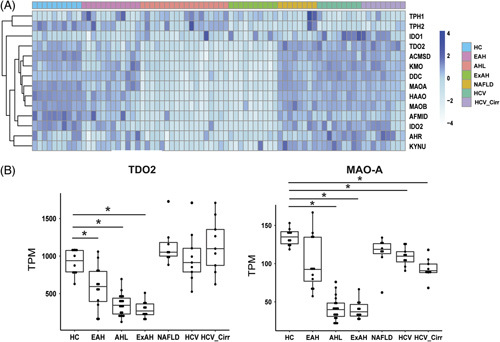
Hepatic gene expression of enzymes involved in tryptophan metabolism is impaired with severe ALD. Heatmaps were created by log transforming transcripts per million and grouping samples by disease categories, such as HC (n = 10), early AH (n = 12), AH with liver failure (n = 18), explant tissue from patients with sAH with emergency liver transplant (ExAH, n = 10), NAFLD (n = 8), HCV (n = 9), and HCV_Cirr (n = 9). (A) Expression of enzymes involved in tryptophan metabolism pathways. (B) Boxplots of TDO2 and MAOA gene expression in transcripts per million from RNA-seq data of patients with various liver pathologies. Error bars indicate SD, and (*) indicates q < 0.05. Abbreviations: AH, alcohol-associated hepatitis; ALD, alcohol-associated liver disease; ExAH, explant tissue from patients with alcohol-associated hepatitis; HC, healthy control; HCV_Cirr, hepatitis C virus with cirrhosis; sAH, severe alcohol-associated hepatitis.

Using the bulk RNA-seq data, we observed that genes involved in tryptophan metabolism were also perturbed in patients with sAH. In the kynurenine metabolism pathway, tryptophan is initially metabolized by enzymes IDO1 and IDO2 along with TDO2, in the rate-limiting first step of the reaction. Not only was expression of IDO2 and TDO2 decreased in the sAH groups but also downstream enzymes within the kynurenine pathway such as kynurenine 3-monooxygenase were also perturbed (Figure 4A). Of these enzymes, TDO2 was most highly expressed in the liver and, interestingly, was also decreased in patients with early AH (Figure 4B). After synthesis in the gut, serotonin can be converted to 5-hydroxyindole acetaldehyde by MAOA in the liver; expression of MAOA was also impaired in severe ALD (Figure 4C).

### Protein expression of enzymes involved in p-Cresol metabolism was decreased in patients with sAH

**FIGURE 5:**
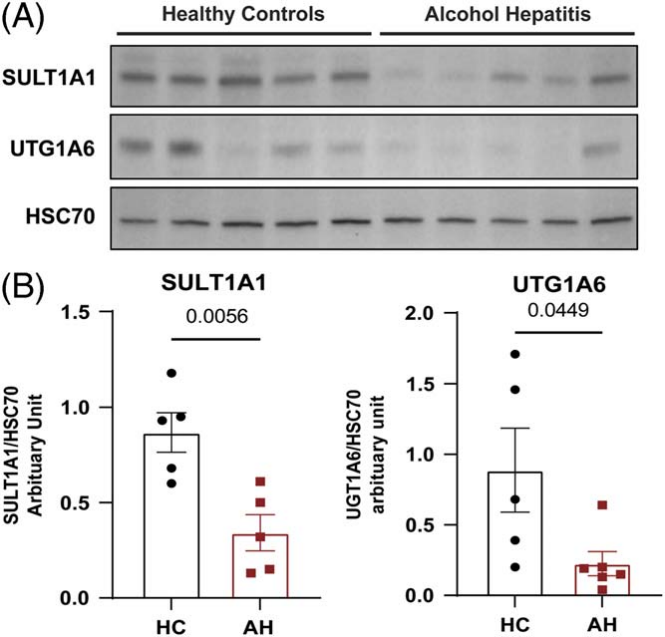
Protein expression of enzymes involved in p-cresol metabolism is decreased in patients with sAH. (A) Liver SULT1A1 and UGT1A6 protein expression measured by western blot from healthy controls and patients with sAH. (B) Densitometric analysis of protein expression for SULT1A1 and UGT1A6 in healthy controls and patients with sAH. Abbreviations: sAH, severe alcohol-associated hepatitis; SULT, sulfotransferase; UGT, UDP glucuronosyltransferase.

In order to validate the results from the bulk RNA-seq analysis, western blot analysis of the 2 enzymes that metabolize p-cresol, UGT1A6 and SULT1A1, was performed in liver tissue samples from HCs and patients with sAH. UGT1A6 is the enzyme responsible for the glucuronidation of p-cresol and the formation of p-cresol glucuronide, while SULT1A1 is the predominant enzyme for the formation of p-cresol sulfate. In agreement with the RNA-seq data, protein expression of both SULT1A1 and UGT1A6 was decreased in patients with sAH compared to HCs (Figure 5).

## Discussion

Through the use of stable isotope dilution liquid chromatography–mass spectrometry,^13^ we quantified circulating levels of multiple structurally specific gut microbiome-produced compounds and uncovered novel dysregulation of microbial metabolites in patients with ALD. Prior research has demonstrated that factors contributing to ALD, such as inflammation and gut dysbiosis, are exacerbated in more severe stages of ALD.^5^ This exacerbated dysbiosis can lead to perturbations of microbial metabolites that enter the circulation to exert their effects on various organs. However, changes in circulating microbe-derived metabolites from patients with ALD have seldom been explored.

In ALD, several gut microbe-derived metabolites that are positively associated with cardiorenal diseases such as p-cresol sulfate were decreased in the circulation of patients with severe forms of ALD. We hypothesized that 1 potential explanation for this unexpected finding was perturbations in the host liver phase II metabolism. Once metabolites, for example p-cresol, are produced by the gut microbiome, they are delivered to the liver through the portal vein, making the liver the primary site of metabolism for these xenometabolites. Phase II metabolism often involves conjugation reactions designed to increase the polarity of compounds in order to facilitate excretion to the urine or the feces. RNA-seq analysis showed that the enzymes involved in glucuronidation and sulfonation were impaired in patients with severe forms of ALD, and the expression of enzymes directly involved in the conjugation of sulfates and glucuronides in this targeted panel was decreased. These findings suggest that alcohol-induced gut dysbiosis may contribute to circulating metabolomics changes, but, in parallel, impaired hepatic metabolism of microbial metabolites could also shape the ALD-associated plasma metabolome.

P-cresol is a gut-derived metabolite produced as a result of bacterial metabolism of tyrosine.^25^ Its conjugated forms, p-cresol sulfate and p-cresol glucuronide, are positively associated with a variety of diseases including cardiovascular disease, chronic kidney disease, and more recently discovered, autism spectrum disorder.^13,29,30^ Patients with these diseases have high levels of meta-organismal sulfates, which can be directly toxic to renal cells; thus, metabolites like p-cresol sulfate, indoxyl sulfate, and 4-ethylphenyl sulfate are considered to be uremic toxins.^30^ However, both conjugated forms of p-cresol can be anti-inflammatory through their actions on TLR4. In a model of allergic airway inflammation, p-cresol sulfate protects against inflammation through its actions on TLR4 signaling, while p-cresol glucuronide promotes blood-brain barrier integrity and acts as a TLR4 antagonist.^31,32^ Indeed, lipopolysaccharide activation of TLR4 signaling is a hallmark feature of ALD, and microbial sulfates could be modulating immune responses through this signaling pathway.^4^

Phase II metabolism encompasses conjugation reactions designed to detoxify and increase the disposal of drugs, xenobiotics, and endogenous compounds.^26^ We find that patients with sAH have decreased expression of glucuronosyltransferases and steroid sulfotransferases, including SULT1A1, SULT1E1, and SULT2A1. Patients with AC also have decreased hepatic protein expression of phase II glucuronidation and sulfonation enzymes,^33,34^ consistent with the lower concentrations of p-cresol conjugates in this population (Figure 1). Interestingly, other less highly expressed sulfotransferases have increased expression, suggesting that the changes in expression of phase II enzymes are not solely due to impaired synthetic capacity of the liver in ALD. The sulfotransferases that were increased in expression involve sulfonation of neurotransmitters, bile acids, and some steroids; more work will be required to understand if their metabolism is disrupted, similarly to gut metabolites. Moreover, 1 consequence of this disruption of phase II metabolism could be increased concentrations of unconjugated p-cresol, which has been shown to display some toxicity to hepatocytes *in vitro*, albeit at extremely high concentrations.^35^ However, 1 limitation in our study is that we were unable to measure unconjugated p-cresol as it is highly volatile and requires appropriate derivatization. Metabolites of tryptophan have been well studied in regard to their ability to modulate immune responses through the activation of the AHR, especially in regard to ALD.^10,12^ Indeed, tryptophan metabolites such as indole-3-propionic acid and serotonin were decreased in severe ALD, with the lowest concentrations being found in patients with sAH. However, less abundant tryptophan metabolites, for example, methyl-indole acetic acid and indoleacrylic acid, were increased in ALD. Indoxyl sulfate is considered both a metabolite of indole, capable of activating AHR and reducing intestinal inflammation, as well as a uremic toxin often implicated as a biomarker for kidney and cardiovascular disease.^30,36^ While these metabolites may display toxicity and positive associations with other diseases, they may be beneficial in alcohol-induced liver injury. Not only does AHR play a role in modulating immune responses but it has also been implicated as a regulator of metabolic pathways, such as phase II metabolism.^37^ Activation of AHR can lead to increased expression of UGT enzymes, while AHR knockout leads to broad alterations in the murine metabolome.^38,39^ Because these core metabolic pathways are closely intertwined, decreased AHR activation could be modulating the expression of phase II enzymes, or vice versa, leading to exacerbated liver injury in ALD.

Given the growing appreciation of how alterations in the gut microbiome are strongly associated with diverse forms of liver disease, there may be untapped therapeutic potential in gut microbiome-targeted therapies. Rational design of gut microbe-targeted therapeutic strategies requires a comprehensive understanding of both gut microbe-driven metabolism and host cometabolism within the human meta-organism. Here, we have provided some initial insights into how both microbe and host aromatic amino acid–derived metabolites associate with ALD. However, additional studies will be required to understand how other structurally diverse metabolites that originate from gut microbes are altered in ALD. With rapidly advancing metabolomics techniques, we are now poised to more comprehensively understand the gut microbe-associated metabolome as it relates to human disease and leverage this into new therapeutic strategies. Several recent examples also show potential therapeutic benefits afforded by gut microbiome-targeted therapies.^11,40–44^ Although drug discovery has historically focused on human target engagement, an exciting time lies ahead where we could instead target the microorganisms that live within us to combat ALD.

## Supplementary Material
