# Supplementary material for: Dysregulated meta-organismal metabolism of aromatic amino acids in alcohol-associated liver disease
Source: Hepatol Commun. 2023 Oct 12;7(11):e0284. doi: 10.1097/HC9.0000000000000284 (PMC10578770; doi:10.1097/HC9.0000000000000284)

Supplementary Figures:

Dysregulated Meta-Organismal Metabolism of Aromatic Amino Acids in Alcohol-Associated Liver Disease

Marko Mrdjen[1,2,3], Emily Huang[1], Annette Bellar[1], Nicole Welch[1,4], Jaividhya Dasarathy[5], David Streem[6], Craig J McClain[7], Mack Mitchell[8], Svetlana Radaeva[9], Bruce Barton[10], Gyongyi Szabo[11], Srinivasan Dasarathy[1,4,12,13], Zeneng Weng[2,12,13], Stanley L Hazen[2,12,13], J. Mark Brown[2,3,12,13], Laura E Nagy[1,12,13]

Supplemental Figure 1 – Concentrations of microbe-derived metabolites

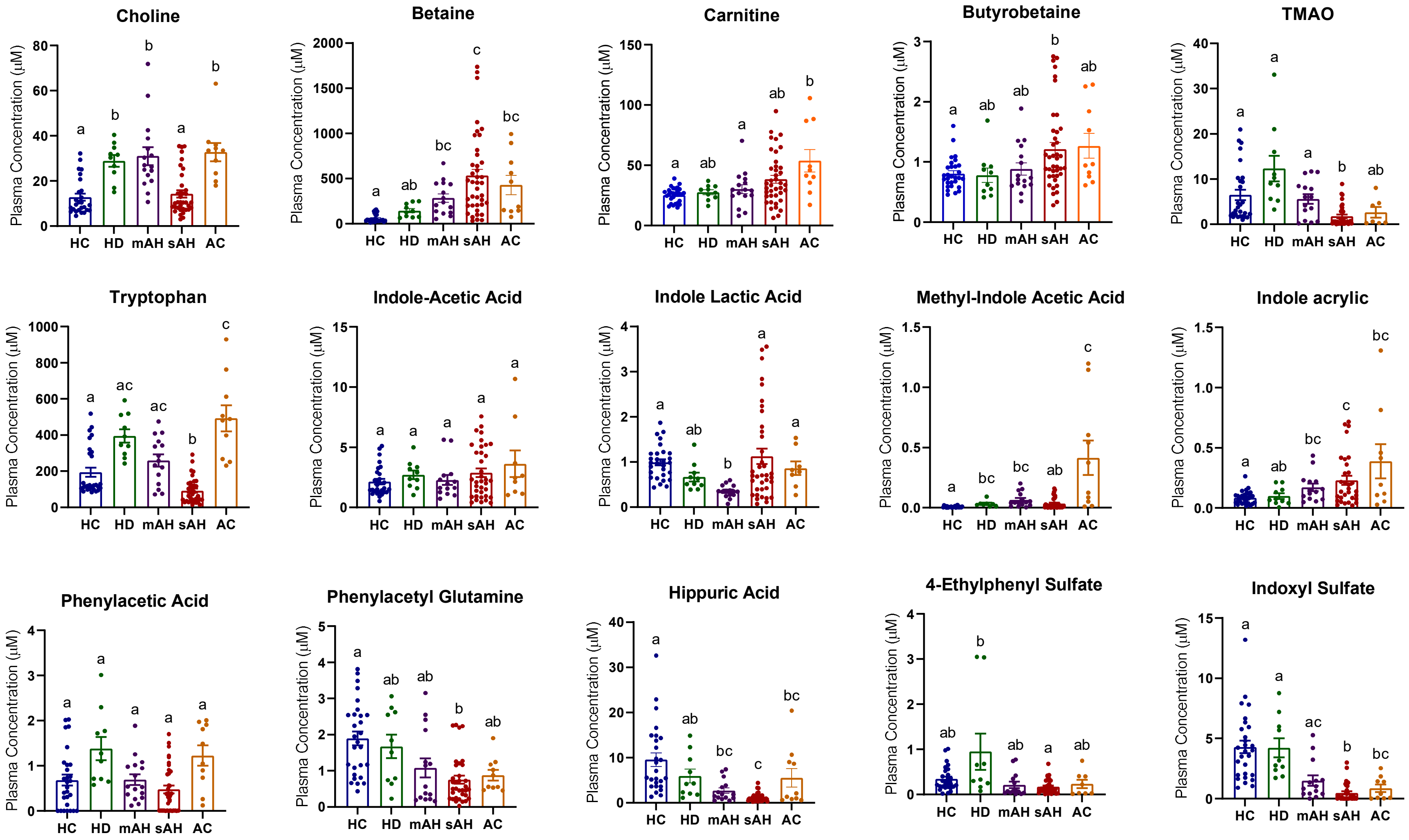

Supplementary Figure 2 – Metabolite correlations with clinical features of sAH and mAH

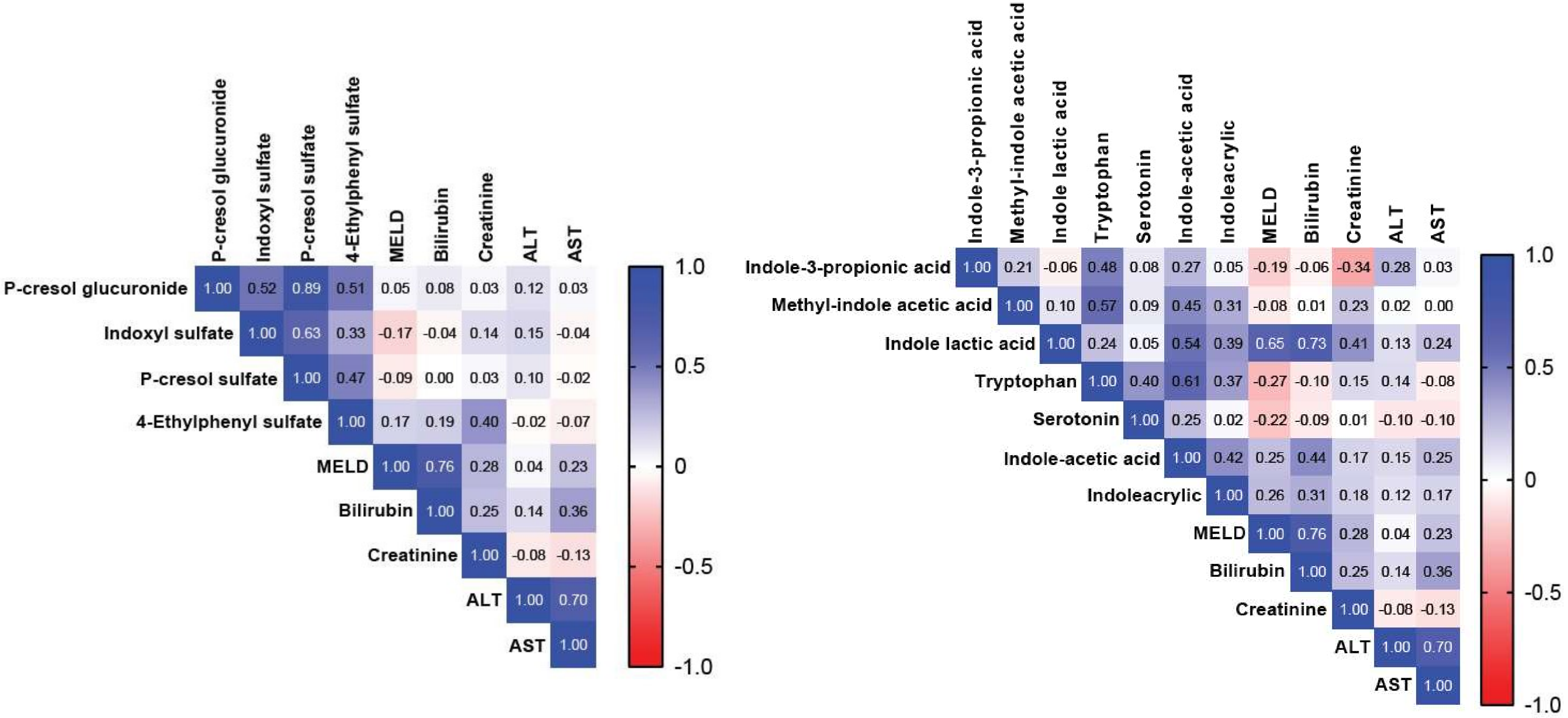

Supplementary Figure 3 – Correlations between metabolites and glomerular filtration rate in patients with sAH

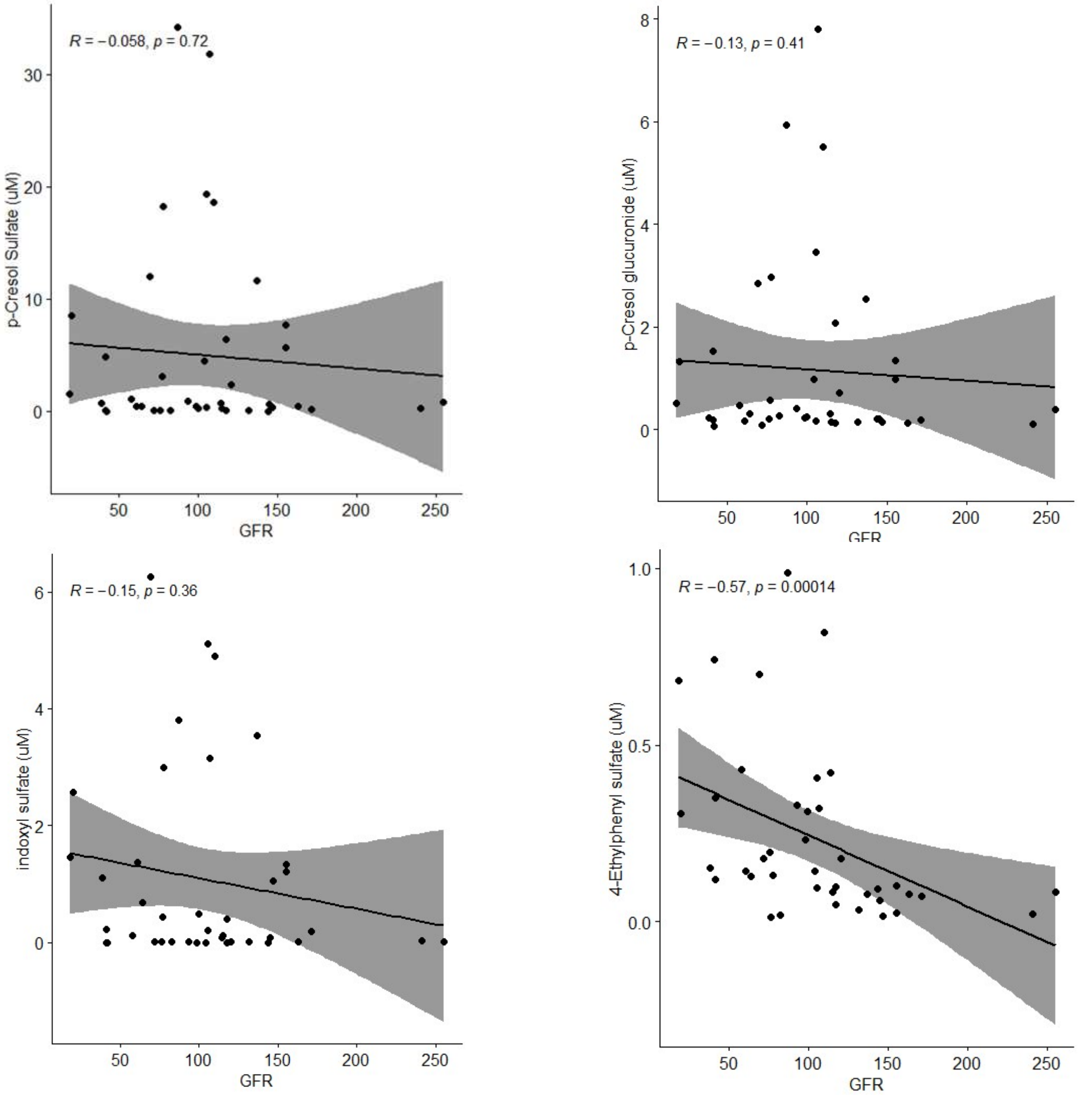

Supplement: Supplementary file 1 [file hc9-7-e0284-s001.pdf]
